# Risk factors for appendiceal involvement in women with epithelial ovarian cancer

**DOI:** 10.4274/jtgga.2017.0005

**Published:** 2017-09-01

**Authors:** Mustafa Erkan Sarı, Elmas Korkmaz, Murat Öz, Tayfun Güngör, Mehmet Mutlu Meydanlı

**Affiliations:** 1 Department of Gynecologic Oncology, Zekai Tahir Burak Women’s Health Training and Research Hospital, University of Health Sciences Faculty of Medicine, Ankara, Turkey

**Keywords:** Epithelial ovarian cancer, appendectomy, risk factors

## Abstract

**Objective:**

To evaluate the risk factors for appendiceal involvement in women with epithelial ovarian cancer (EOC) who underwent appendectomy at the time of initial surgery.

**Material and Methods:**

Patients with a final diagnosis of EOC who underwent appendectomy at the time of initial surgery were evaluated retrospectively. Risk factors related to the presence of appendiceal involvement were analyzed.

**Results:**

A total of 210 patients underwent appendectomy during staging surgery. Appendiceal involvement was detected in 61 patients. No women with apparent clinical early-stage tumors had evidence of isolated metastatic disease to the appendix; therefore, no upstaging was detected due to solitary appendiceal involvement in this group of patients. For all patients, univariate analysis of the appendiceal involvement revealed age, stage, grade, extragenital organ involvement (omentum, bowel, peritoneum), positive cytology, and lymph node metastasis as significant factors (p<0.05). In the multivariate analysis, appendiceal involvement was significantly affected by age and omental involvement. Older age (>50 years) [odds ratio (OR) 2.8; 95% confidence interval (CI): (1.24-6.37); p=0.014] and presence of omental involvement [OR: 3.2; 95% CI: (1.22-8.59); p=0.018) seemed to be independent risk factors for appendiceal involvement in women with EOC.

**Conclusion:**

Our findings indicate that routine appendectomy at the time of surgery for apparent early-stage EOC is not warranted. Nevertheless, the surgeon can take the initiative in regards to performing appendectomy because the morbidity rates due to this procedure are negligible. Older age (>50 years) and presence of omental involvement seem to increase the risk of appendiceal involvement by 2.8 and 3.2 times, respectively.

## INTRODUCTION

The 2014 statements of International Federation of Gynecology and Obstetrics (FIGO) recommends surgical staging for epithelial ovarian cancer (EOC) based on findings during exploration (1). The standard staging procedure for EOC includes exploratory laparotomy, extrafascial hysterectomy, bilateral salpingo-oophorectomy, pelvic and para-aortic lymph node (LN) dissection, ascites sampling/peritoneal washing, multiple peritoneal biopsies, and omentectomy.

It has been reported that the appendix was a potential site of involvement in patients with EOC (2). The presence of solitary appendiceal involvement upstages the disease to stage III. Therefore, appendectomy can be added to the staging surgery for both accurate staging and optimal cytoreduction. The majority of studies do not routinely recommend appendectomy in clinical early stage disease, i.e. stage I and II. The only indication for routine appendectomy is in patients with mucinous histology, even in early stages, in order to exclude a possible appendiceal carcinoma. However, routine removal of the appendix in other histologiesis also recommended by some authors so as to achieve complete staging and cytoreduction (3, 4).

In the present study, we aimed to evaluate the risk factors for appendiceal involvement in patients with EOC who underwent appendectomy during staging surgery by analyzing the histopathologic findings in appendectomy specimens. Complications related to appendectomy were also assessed.

## MATERIAL AND METHODS

Following the institutional review board approval, the pathological reports, medical records, and operation notes of patients who underwent staging surgery with a pre-diagnosis of EOC between January 2008 and December 2013 were retrospectively evaluated. All patients provided informed consent regarding the research and use of their medical information at admission.

All patients were staged according to the FIGO staging system for ovarian carcinoma. Data regarding age, menopausal status, disease stage, grade, histologic subtype, cytology, status of extragenital organ involvement, LN metastasis and complications related to appendectomy (e.g., peri-appendiceal abscess, intestinal obstruction, and peritonitis) were extracted from the records. Patients whose final pathology was any benign disease, primary appendiceal carcinoma, primary peritoneal cancer or metastatic disease were excluded. Appendiceal involvement was considered microscopic if the appendix was noted to be grossly normal by the operating surgeon and pathologist but histologic sections were positive for disease. If the appendix was observed abnormal during the surgery, the involvement was considered gross.

The primary end point of the present study was the determination of risk factors for appendiceal involvement in women with EOC.

Statistical analyses were performed using the Statistical Package for the Social Sciences software (version 18, SPSS, Inc. Chicago, IL, USA). Data are expressed as median and range for continuous variables and binary variables are reported as counts and percentages. A simple logistic regression analysis was performed to determine the correlation of patient and tumor characteristics with appendiceal involvement. A p value <0.05 was considered to indicate statistical significance. Variables with a p value less than 0.05 were included in the multiple logistic regression analysis. The impact of each factor on appendiceal metastasis was evaluated.

## RESULTS

A total of 210 patients with a final diagnosis of EOC who underwent appendectomy at the time of initial surgery were evaluated retrospectively. The median age of the patients was 51 years (range, 28-81 years). Patient and disease characteristics are shown in Table 1.

All patients with appendiceal involvement also had extra-pelvic disease, which already upstaged them to stage III or IV. The histopathologic results of patients who had appendiceal involvement are shown in Table 2. No evidence of macroscopic or microscopic metastasis in the appendix was observed in patients with early-stage EOC during intraoperative and postoperative evaluation. The rate of appendiceal involvement was significantly higher in patients with advanced-stage disease. For the entire cohort, univariate analysis revealed age, stage, grade, extra-genital organ involvement, positive cytology, and LN metastasis as significant risk factors for appendiceal involvement. However, no correlation was found between histopathology and appendiceal involvement. Univariate analysis of risk factors associated with appendiceal involvement in women with EOC (n=210) is shown in Table 2.

When women with advanced-stage disease were evaluated alone, age, presence of positive cytology, and involvement of the omentum and bowel were detected as significant factors via univariate analysis; however, stage, grade, histopathology, LN metastasis, and other organ involvement had no effect on appendiceal involvement (Table 3).

In the multivariate analysis, older age (>50 years) [odds ratio (OR): 2.8; 95% confidence interval (CI): (1.24-6.37); p=0.014] and the presence of omental involvement [OR: 3.2; 95% CI: (1.22-8.59); p=0.018] were found as independent risk factors for appendiceal involvement in women with EOC (Tables 4 and 5).

No intraoperative or postoperative complications directly related with appendectomy were detected.

## DISCUSSION

The main target in the treatment for EOC is accurate surgical staging and maximal cytoreduction. This procedure is extremely important and necessary in order to increase the rates of disease control and survival.

Ovarian cancer initially spreads inside the abdominopelvic cavity. Although patients with clinically early-stage disease rarely tend to have appendiceal involvement, evaluation of the appendix is necessary during surgery because appendiceal involvement leads to upstaging and demands adjuvant treatment. The rate of appendiceal metastasis has been shown to be low; therefore, routine performance of appendectomy in women with early-stage EOC is still controversial. Routine performance of appendectomy is justified by some authors in all stages of EOC in order to achieve complete staging (3, 4). On the contrary, other studies suggested that appendectomy was not warranted in early-stage EOC because the risk of involvement was extremely low (2, 5, 6, 7, 8).

Westermann et al. (2) retrospectively evaluated the results of 53 patients with EOC who underwent appendectomy during staging surgery and reported the rate of appendiceal involvement as 34%. Four of these patients had normal appendixes at macroscopic evaluation. However, it is impossible to assess the actual rate of appendiceal involvement in early-stage disease because the authors did not mention the stage of the patients.

The first study that reported an appendiceal involvement rate and made a distinction between early and advanced-stage disease was published by Malfetano (5). In that study, the rate of appendiceal involvement was 51% and 70% in all patients and in women with advanced-stage disease, respectively. Appendiceal involvement did not lead to upstaging in any patients with early-stage disease, and was not the solitary site of involvement in women with stage III disease. Fontanelli et al. (6) reported the rate of appendiceal involvement as 23% in 160 patients with EOC. The authors stated that 91% of these 23 patients had serous histology and grade 2-3 disease, adding that no appendiceal involvement was observed in early-stage EOC. Ramirez et al. (7) reported the results of 57 patients with stage I-II ovarian cancer and stated that no appendiceal involvement or appendectomy-related complications were observed. The largest study on appendiceal involvement in EOC was published by Lee et al. (9) which reported the results of 149 women who had no clinical disease outside the pelvis. They reported no appendiceal involvement or upstaging due to isolated appendiceal involvement in these patients. As a conclusion of these studies, routine performance of appendectomy is not recommended in patients with stage I-II EOC.

On the other hand, there are two studies that reported microscopic appendiceal involvement in patients with apparent early-stage EOC. The first study was reported by Rose et al. (3) who performed appendectomy in 80 patients during staging surgery and found the appendiceal involvement rate as 31%. Nevertheless, they stated that in a total of 47 patients with early-stage disease, microscopic involvement of the appendix was detected in 2 (4.3%) patients, but added that these patients already had stage III disease due to omental involvement. None of the patients with early-stage disease were upstaged due to isolated appendiceal involvement; however, appendiceal involvement was detected in 70% of patients who already had advanced-stage disease. In addition, the appendiceal involvement rate was higher in patients with serous carcinoma compared with those with mucinous carcinoma (48% vs. 8%, respectively). Although the results of this study were consistent with earlier series, the authors recommended routinely removing the appendix owing to the low morbidity rate related to appendectomy. Subsequently, the authors reported a case with isolated microscopic appendiceal involvement in a patient with clinical early-stage EOC (10). The second study that recommended the routine removal of the appendix was published by Ayhan et al. (4). In that study, the rate of appendiceal involvement was reported as 9.8% in 102 patients with clinical stage I-II disease. In a total of 10 patients with microscopic appendiceal involvement, 5 patients were upstaged due to this finding. The overall rate of appendiceal involvement was reported as 37% and the rate of upstaging due to isolated appendiceal involvement was 5% in patients with clinical early-stage disease. Therefore, the authors recommended that routine appendectomy should be performed during staging surgery in all patients with EOC, even if they have clinical early-stage disease.

In the present study, 210 women who underwent appendectomy at initial surgery were evaluated. None of the patients with an apparent clinical early-stage tumor had evidence of isolated appendiceal involvement; as such, upstaging due to solitary involvement of the appendix was not detected in this group of patients, consistent with the results of earlier series.

Nevertheless, routine appendectomy is recommended particularly in patients with ovarian mucinous cystadenocarcinoma owing to the fact that it is frequently observed as a metastatic tumor from the gastrointestinal system in which the appendix may contain the primary lesion (11, 12). However, the rate of appendiceal involvement was 18.7% in women with mucinous cystadenocarcinoma in the current study. The relatively low rate of appendiceal involvement in mucinous histology can be attributed to the limited number of patients with mucinous histology (n=18) in our cohort. Although our findings do not completely support the concept of routine performance of appendectomy in women with mucinous EOC, appendectomy should be a routine component of comprehensive surgical staging in all patients with mucinous histology based on the evidence in the literature. However, there are also studies that reported higher rates of appendiceal involvement in patients with advanced-stage serous adenocarcinoma (3, 6, 13).

Tumor grade was detected as a prognostic factor for appendiceal involvement. In the study by Ayhan et al. (4) the rates of appendiceal involvement in women with grade 1, 2, and 3 disease was reported as 18.2%, 33.8%, and 48.2%, respectively. However, grade was not detected as a significant factor for appendiceal involvement via multivariate analysis, stage being the only factor that was statistically significant. Furthermore, patients with grade 2 and 3 EOC had a higher rate of appendiceal involvement in the study by Malfetano (5).

In the present study, univariate analysis revealed age, stage, grade, extra-genital organ involvement, presence of positive cytology, and LN metastasis as significant factors for appendiceal involvement in all patients, but histopathology was not found to be a risk factor for the involvement of the appendix. When patients with advanced-stage disease were evaluated alone, age, presence of positive cytology, and involvement of the omentum and bowel were found as significant factors in univariate analysis, but stage, grade, histopathology, LN metastasis, and other organ involvement had no effect on the risk of appendiceal involvement. In the multivariate analysis, the risk of appendiceal involvement was significantly affected by age and omental involvement. In addition to these findings, we observed no complications directly related with appendectomy in the present study.

Although this study represents one of the largest series on the evaluation of appendiceal involvement in EOC, the retrospective design of this study may be a significant source of bias. However, to our knowledge, this is the first study in the literature to mention omental involvement as a risk factor for appendiceal involvement in EOC.

The only definitive indication for appendectomy in early-stage EOC seems to be mucinous histopathology in order to exclude ovarian metastasis of primary appendiceal carcinoma in light of data in the literature.

## CONCLUSION

Our findings indicate that routine appendectomy at the time of surgery for apparent early-stage EOC is not warranted. Nevertheless, surgeons can take the initiative in regards to performing appendectomy because the morbidity rate due to this procedure is negligible. Older age (>50 years) and the presence of omental involvement seem to increase the risk of appendiceal involvement in EOC.

## Figures and Tables

**Table 1:**
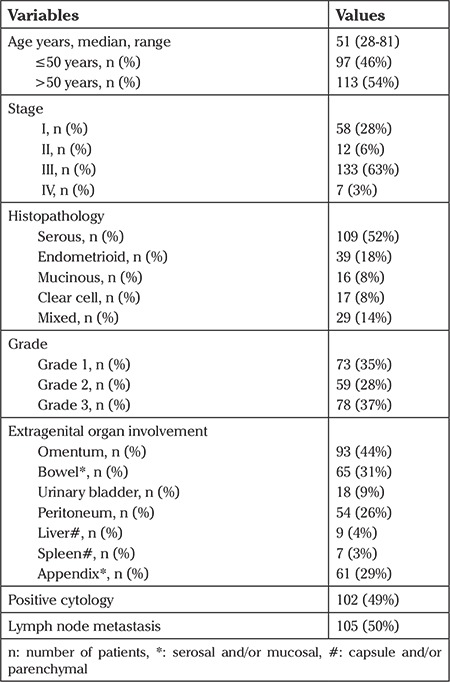
Results of patients who underwent appendectomy at the time of initial surgery

**Table 2:**
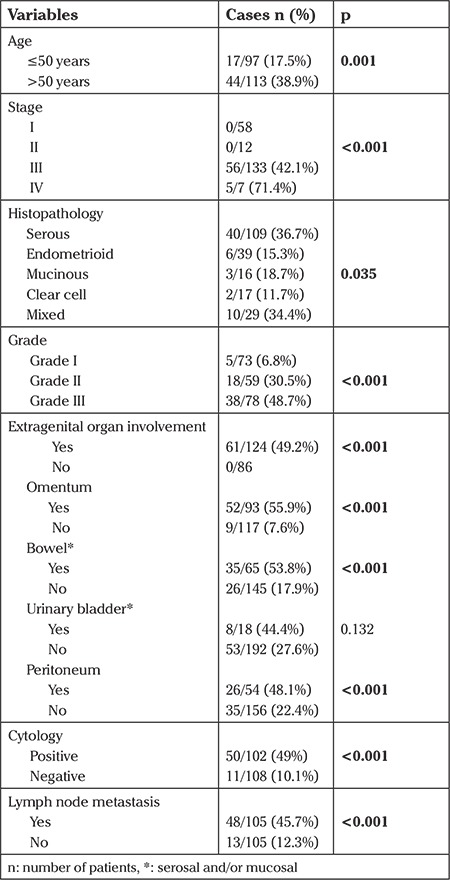
Univariate analysis of risk factors for appendiceal involvement in women with epithelial ovarian cancer (n=210)

**Table 3:**
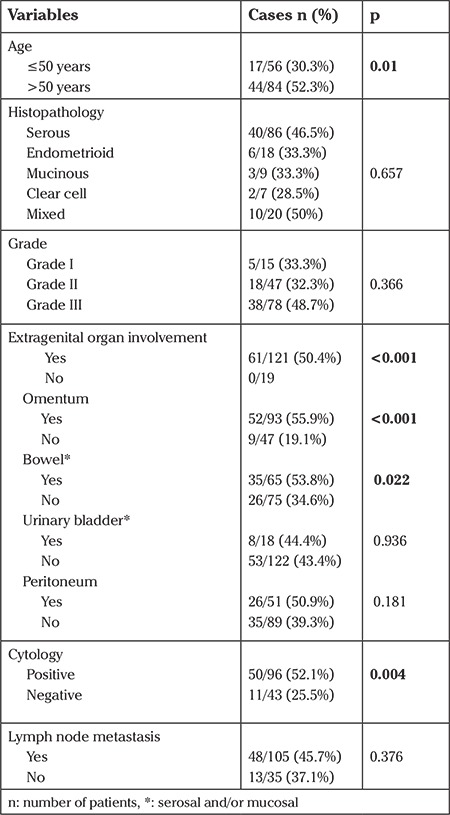
Univariate analysis of risk factors for appendiceal involvement in women with advanced-stage epithelial ovarian cancer (n=140)

**Table 4:**
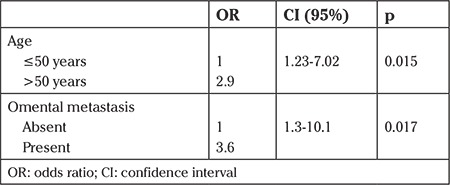
Multivariate analysis of risk factors for appendiceal involvement in women with epithelial ovarian cancer (n=210)

**Table 5:**
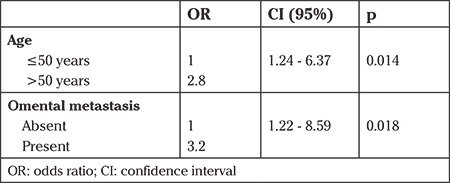
Multivariate analysis of risk factors for appendiceal involvement in women with advanced-stage epithelial ovarian cancer (n=140)
